# Authorization and Toxicity of Veterinary Drugs and Plant Protection Products: Residues of the Active Ingredients in Food and Feed and Toxicity Problems Related to Adjuvants

**DOI:** 10.3389/fvets.2017.00146

**Published:** 2017-09-04

**Authors:** Szandra Klátyik, Péter Bohus, Béla Darvas, András Székács

**Affiliations:** ^1^Agro-Environmental Research Institute, National Agricultural Research and Innovation Centre, Budapest, Hungary; ^2^Lamberti S.p.A., Albizzate, Italy

**Keywords:** veterinary drugs, pesticides, active ingredients, additives, adjuvants, surfactants, ecotoxicity

## Abstract

Chemical substances applied in animal husbandry or veterinary medicine and in crop protection represent substantial environmental loads, and their residues occur in food and feed products. Product approval is governed differently in these two sectors in the European Union (EU), and the occurrence of veterinary drug (VD) and pesticide residues indicated by contamination notification cases in the Rapid Alert System for Food and Feed of the EU also show characteristic differences. While the initial high numbers of VD residues reported in 2002 were successfully suppressed to less than 100 cases annually by 2006 and on, the number of notification cases for pesticide residues showed a gradual increase from a low (approximately 50 cases annually) initial level until 2005 to more than 250 cases annually after 2009, with a halt occurring only in 2016. Main notifiers of VD residues include Germany, Belgium, the UK, and Italy (63, 59, 42, and 31 notifications announced, respectively), and main consigning countries of non-compliances are Vietnam, India, China, and Brazil (88, 50, 34, and 23 notifications, respectively). Thus, countries of South and Southeast Asia are considered a vulnerable point with regard to VD residues entering the EU market. Unintended side effects of VDs and plant protection products may be caused not only by the active ingredients but also by various additives in these preparations. Adjuvants (e.g., surfactants) and other co-formulants used in therapeutic agents and feed additives, as well as in pesticide formulations have long been considered as inactive ingredients in the aspects of the required main biological effect of the pharmaceutical or pesticide, and in turn, legal regulations of the approval and marketing of these additives specified significantly less stringent risk assessment requirements, than those specified for the active ingredients. However, numerous studies have shown additive, synergistic, or antagonistic side effects between the active ingredients and their additives in formulated products; moreover, toxicity has been evidenced for various additives. Therefore, toxicological evaluation of surfactants and other additives is essential for proper environmental risk assessment of formulations used in agriculture including animal husbandry and plant protection.

## Introduction

Large quantities of various chemical compounds and their formulations are used in several fields of agriculture, such as veterinary medicine, animal husbandry, animal nutrition, and chemical plant protection, and these substances may have adverse effects on the environment. Food/feed and environmental safety of these formulated products are governed by several approaches.

Active ingredients of both veterinary drugs (VDs) and plant protection products (PPPs), i.e., pesticides are strictly regulated in the European Union (EU) regarding both their approved use and allowed level of occurrence in animal products. As for approval for use, the active ingredients are registered at EU level, while authorization of the products is carried out at EU or at Member State (MS) level. Such a dual registration protocol has certain, clear benefits, e.g., the formulated products are approved according to regional needs (ecological considerations—biogeographical regions) and also results in disadvantages (e.g., regulatory rigidity as given problems with the formulated products may not be addressed at EU level, but have to be dealt with by each MS). As for post-market monitoring, maximum residue limits (MRLs) for the active ingredients and their metabolites are defined by law in both sectors (VDs and PPPs) and are subject to official monitoring by the competent authorities, facilitated by the Rapid Alert System for Food and Feed (RASFF) of the EU.

The applied formulations may contain various additives (e.g., surfactants), besides the active ingredients, and these additives have long been classified as inert or inactive components in the aspect of the main biological effects of the formulation. Despite their name, however, inert ingredients may be biologically or chemically active in their side effect profile and are labeled as inert only because of their function in the formulated product.

## Legal Regulations for the Registration of VDs and Pesticides

Authorization and distribution of agrochemicals are strictly regulated worldwide. Although these regulatory frameworks for VDs and PPPs have different historic origins, the former having roots in the legal regulations of human pharmaceuticals, similarities, and characteristic differences exist between these two sectors. Important similarity aspects include the legal approval systems being focused on scientific evidence-based risk assessment (RA) and putting a strong emphasis on safety, primarily toward improving human health (1). Possible direct or indirect environmental risks have received increasing attention lately in both groups, yet regulatory pharmacology and toxicology of VDs are more pronouncedly oriented by a comparative medicine aspect, then the assessment of PPPs.

### Veterinary Drugs

Extensive control of VDs is required in the EU, and thus, the requirements are very strict not only for quality and efficacy but also for safety, including animal and human health and environmental risk assessment (ERA), similarly to the assessment and regulation of human medicines. Upon revision, veterinary legislation Directives 81/851/EEC and 81/852/EEC (2, 3) were amended by Directives (EEC) 2004/28 and 2009/9 (4, 5). Specific directives and legal specifications regulate the distribution and required quality of veterinary substances, including veterinary medical products, ready-made veterinary products, blood products, and homeopathic preparations (2, 6, 7), while immunological veterinary medical products, medicated feeding stuffs and pre-mixes, and biocidal products used for veterinary hygiene are regulated elsewhere. In the EU, two main processes are available for authorizing veterinary medicines: a centralized EU procedure and national protocols. In the centralized procedure, medicinal products are authorized at EU level by the European Medicines Agency (EMA), established in 2004 (6). At national levels, medicines are authorized by MSs in their own territory on the basis of either their own RA or RA carried out in another MS if accepted on the basis of mutual recognition or the decentralized procedure (4, 7). The conditions of marketing authorizations for medicinal products for human and veterinary use are set by Regulation (EC) 712/2012 amending Regulation (EC) 1234/2008 (8, 9). The health RA and ERA requirements of veterinary pharmaceuticals include and ensure the safety of the patient, the user, the products used for food producing animals, the consumers, and the environment, as well. The major aspects of health RA and ERA are quality (e.g., composition, stability, and shelf-life), safety [e.g., consumer safety and residues (only for food producing animals), user, patient, and environmental safety], and efficacy (e.g., pharmacodynamics, pharmacokinetics, laboratory studies, and clinical trials). RA of VDs is carried out on a continuous basis also upon the approved commercial distribution of the preparations, and product quality, efficacy, and safety are routinely monitored by the regulatory and monitoring authorities (1). Pharmaceuticals used in VDs are tested on target species at the therapeutic dose and at its multiples. MRLs for VDs are set by Regulation (EC) 470/2009 (10) that replaced and repealed Regulation (EEC) 2377/90, introducing number of modifications and improvements (11). The regulation of MRLs for VDs includes any ingredients used in veterinary pharmaceuticals and vaccines with pharmacological or pharmacodynamic activity; therefore, evaluation of stabilizers, antioxidants, solvents, and coloring agents is also required. The overall purpose is to ensure the protection of consumers from potentially harmful drug residues in food of animal origin. Pharmacovigilance is an integral part of the regulation for both veterinary and human medicines in the EU, used to describe the collection of information on the adverse effects of pharmaceutical agents (12).

### Plant Protection Products

Plant protection products are governed in the EU by Regulation 1107/2009 (EC), the “Pesticide Act” (13). A rather important feature of the pesticide registration policy is that pesticide active ingredients are authorized at the EU level, while formulated PPPs and their uses on given crop commodities are registered at MS level. The active ingredients must be approved for use by the European Commission (EC) to be considered for being marketed in any form of pesticide formulations. In the process of authorization, these substances are evaluated in scientific evidence-based RA by the European Food Safety Authority (EFSA), established in 2002 (14). RA statements issued by EFSA, debated, and commented by the MSs are the basis of the subsequent EC decisions regarding authorization. Active ingredients classified as carcinogenic, mutagenic, teratogenic, endocrine disruptor, persistent, and bioaccumulative substances cannot be approved (15). Pesticide active ingredients regularly undergo detailed reassessment, and during the last major re-registration process, completed in 2010, the number of the registered active ingredients has substantially been reduced from 959 to approximately 480 compounds authorized now as pesticide active ingredients in PPPs (16).

In contrast to pesticide active substances, formulated PPPs are authorized by the MSs on their territory, in accordance with the corresponding EU rules and regulations. Moreover, the enabled use of the pesticide formulations in various crop cultures is determined at MS level, as well.

To avoid over-excessive human exposure to pesticide residues through foodstuff and the drinking water, MRLs have been established for these compounds in different commodities throughout the world, including the EU, and the levels of pesticide residues are required to be regularly monitored. MRL values are set by the EC for all food and animal feed categories on the basis of a complete RA by EFSA (17). If the levels of residues in case of approved pesticides exceed the determined MRLs in the food and animal feed products, measures have to be taken to prevent the use of the contaminated products/crops. In contrast, previously permitted, but later withdrawn or banned active ingredients of pesticides or their metabolites cannot be present in the food or animal feed at any concentration. These contaminants are usually originated from inappropriate technology or earlier environmental contamination. The official MRLs of pesticide residues are specified in *Codex Alimentarius* (18) and other declarations (17, 19) for various commodities.

As mentioned earlier, PPPs as pesticide formulations are subject to dual approval: registration of their active ingredients at EU level and authorization of the formulated product at MS level. Both levels rely on the determination of physico-chemical, toxicological, and ecotoxicological properties of the substances (the active ingredient or its mixture with its adjuvants), and data determined are used in scientific evidence-based ERA on the basis of both the Pesticide Act and Regulation 1907/2006 (EC), the Registration, Evaluation, Authorization and Restriction of Chemicals (REACH) Act, supervised by the European Chemicals Agency (ECHA), established in 2006 (20). The legal framework for the authorization of feed additives and biocides (falling outside the main scope of this paper) substantially differs from the legal regulation of PPPs. Regulation (EC) No 1831/2003 on additives for use in animal nutrition (21) regulates the placing on the market and use of feed additives and premixtures, including their supervision and labeling. The EU Register of Feed Additives (22) compiled on the basis of this regulation lists numerous types of additives, including emulsifying and stabilizing agents, binding, anti-caking agents and coagulants, preservatives, antioxidants, acidity regulators, enzymes, digestibility enhancers, gut flora stabilizers, coccidostats and histomonostats, microorganisms, silage additives, mycotoxin binders, colorant and flavoring compounds, carotenoids and xanthophyllsm, (pro)vitamins, amino acids, and trace elements. Regulation (EU) 528/2012 the Biocidal Product Regulation (23) concerns the placing on the market and use of biocidal products used to protect humans, animals, materials, or articles against harmful organisms, e.g., pests or bacteria, by the action of the active substances contained in the biocidal product. Although the current paper focuses on VDs and PPPs and does not intend to discuss these two additional groups of products (feed additives and biocides), it has to be noted that given active ingredients may be subject to different legal requirements, when used as VDs (assessed by EMA), “hygienic substances” (biocides) (assessed by EMA or ECHA), or PPPs (assessed by EFSA), which remains a residue contradiction of the current legal setup in the EU (24). In addition, certain toxicity tests required to register PPPs are often performed with the active ingredient alone, not with the pesticide formulation itself. Moreover, ingredients inert in the main effect of the preparation are generally not even indicated on product labels and are often claimed to be confidential business information. This is an improper practice, as “inert” ingredients can significantly affect toxicity endpoints, including developmental neurotoxicity, genotoxicity and disruption of neuroendocrine functions. This phenomenon remains to be another major contradiction in the scope of the legal regulations of pesticides and other biologically active substances (biocides).

### Registration Requirements for Formulation Additives

On the basis of the current legislation, substantially simpler ERA is sufficient for these substances compared to the active ingredients. For example, specific product characteristics (SPC) have to be specified for all components, but the exact percentage quantity of the formulation additive is not required to be specified as public information. SPC has to be quantitatively stated for active ingredients, but the exact content of formulation additives can be specified as proprietary information released only to the registration authorities as classified information in the products documentation. Nonetheless the ERA is specified for formulation additives, as well. The main steps of ERA, similar to the assessment of active ingredients, are hazard identification (e.g., chemical structure and physico-chemical properties), assessment of the exposure [determination of the predicted environmental concentration (PEC), biodegradability assessment] and the effects [acute and chronic toxicity, sub-lethal effects, determination of the predicted no-effect concentration (PNEC)], as well as characterization of the risk on the basis of the ratio of PEC and PNEC (25, 26). The conditions of the ERA are determined by Regulation (EEC) 793/93, Directive (EEC) 93/67, and Regulation (EC) 1488/94 (27–29). The conditions of the authorization and commercial distribution of surfactants (e.g., detergents) in the EU are set by Regulation 648/2004 (EC), adopted on March 31, 2004, and came into force on October 8, 2005 (30), but it focuses primarily on general-purpose surfactants used in laundry detergents and cleaning supplies. As for surfactants in laundry detergents and cleaning supplies, requisites for anionic and non-ionic surfactants regarding primary biodegradability are set in the regulation. Moreover, on the basis of the safeguard clause, if a given surfactant (e.g., detergent) is considered as a risk to human or animal health safety or to the environment by one of the MSs, temporarily special conditions or the proscription of the commercial distribution of the products containing the adverse component can be applied on the area of the given MS. However, RA applies only for surfactants used in laundry detergents and cleaning supplies, and requirements are not as strict as those for biologically active ingredients.

With the introduction of the legal framework of the REACH Act, the EU regulatory system became stricter, and scientific evidence-based RA has been set as a legal requirement to commercialized chemicals (4, 20). Moreover, due to the recognized potential increased toxicity of chemical mixtures, compared to their individual components, the classification, labeling, and packaging of chemical mixtures (e.g., detergents) are specifically regulated by law in the EU (15); and health RA and ERA of additives (e.g., detergents) became substantially more compliant with the RA of the active ingredients.

Currently, the exact chemical name and quantity is legally required to be indicated on the labels of pesticide formulations in the EU only for the active ingredient(s), synergists, and antidotes; therefore, the exact composition and information about adjuvants is not public.

## Safety Assessment of the Active Ingredients

Safety assessment of agrochemicals is an issue of emphasized importance worldwide. The establishment of the food and feed control system at EU level started in 2002 with Regulation (EC) 178/2002 laying down the general principles and requirements of food law, establishing EFSA and laying down procedures in matters of food safety (14). This was followed by a set of regulations on hygiene (31–33), and then Regulation (EC) 882/2004 on official controls performed to ensure the verification of compliance with feed and food law, animal health and animal welfare rules (34). The former separated units, independent authorities, and institutes adopted the food production, trade, and consumption chain approach covering the entire food chain from the farm to the table and enhancing follow-up and prevention. These regulations—to assure high level health and consumer protection—established a new, prevention approach in the food/feed policy. The aim of both the legislative and the advisory systems was utilization of an integrated, “from farm to fork” approach, covering the overall food chain including feed production, primary food production, processing, storage, transport, and trade.

### The EU RASFF

National food safety authorities of the MSs of the EU officially monitor agricultural produce, as well as food and feed commodities for compliance with the current official MRLs of residues of agrochemicals, including VDs and PPPs. To facilitate information exchange among MSs and to the public, RASFF was established in the EU in 1979 (35, 36). RASFF operates in all EU MSs through their national food safety authorities. The system operates on the basis of authority statements on execution measures of the alert system for food and feed safety. Within the system, MSs report to the EC, without delay, any hazards affecting animal and human health directly or indirectly originated from food and feed products or commodities that have been identified through RASFF. The system, operated by the EC, establishes a direct contact among the EC, EFSA, and relevant authorities of the MSs. Any identified hazard related to food and feed and reported to the EC is promptly transferred to all RASFF members. To date, RASFF has been proven to be an effective instrument to exchange information in real time within EU MSs. RASFF is a prominent device to report non-compliances in agricultural commodities and food products with food/feed safety regulations to ensure a direct and real-time exchange of information among countries in the EU and to assist sustenance of an outstanding food/feed safety status.

Data are submitted to the RASFF by National Reference Laboratories (NRLs) in each EU MS and contributing countries. NRLs for the detection of residues are listed in Commission Decision 98/536/EC (37) and Implementing Decisions that followed it, including the latest Regulation 2017/625 (EU), which is the new legal framework for control in food, feed, animal, and plant health (38). High-quality and uniform testing operation of the NRLs is ensured by EU Reference Laboratories (EURLs) governed by Regulation (EC) No 882/2004 on official controls regarding tasks, duties and requirements (34). The EURLs provide NRLs with analytical methods and diagnostic techniques, coordinate their application, train NRL staff, provide the EC with scientific and technical expertise in relation to laboratory analysis, and collaborate with the competent laboratories in non-EU countries. This concerted action of the reference laboratories at EU and national levels assures continuous improvements in the detection capabilities and accuracy within RASFF. Additional sources of improvements in the analytical performance include the introduction of new detection techniques within the range of tools used by NRLs, on one hand, and the expansion of the EU MSs, on the other hand. Advances achieved in method development in food analysis are implemented among the qualitative and quantitative screening and confirmatory tests used at NRLs, and in addition to spectroscopic and chromatographic instrumental methods, functional and biochemical assays, immunoanalytical techniques (immunoassays, immunosensors) and nanoparticle analysis (39) are assessed to expand the range of available methods in food analysis for competent authorities. Moreover, “foodomics” (40), high-throughput analysis (41), and “big data” analysis (42) are also implemented to facilitate food safety. Such analytical progress results in not only the expansion of analysis capacities but also increasing analytical sensitivities and lower limits of detection. Similarly, the enlargement of the EU through the accession of new MSs broadens the residue analysis laboratory network under the Directives 96/23/EC (43) and 98/536/EC (37) reporting to RASFF, improving both its analysis capacity and overall accuracy.

The evolving organization of laboratories involved in the activities of RASFF explains the evolution of the number of contamination cases reported in RASFF. The EC established RASFF with the aim to identify and publicize products on the food market and their producers, distributors violating food/feed safety requirements (44). The database containing analytical results was officially established in 2002 (14), but preliminary data are reported since 1998. Analytical instrumentation of sufficient limits of detection and sample capacity has become available since 2003, since when annual fluctuations are trustworthy. Moreover, analytical determinations have been accompanied by RA since 2011 (36), the decisional system of which is becoming stabilized only gradually, generally becoming stricter.

Searchable databases, summarizing nearly 47 thousand notifications reported until now, 34% of which corresponding to the period of 2012–2016, available at the official Internet portal of RASFF, reflect the current state of imported food/feed commodities in the EU (45), although the overall number of samples analyzed annually, which could provide a view on the real significance of a given problem, is not specified. It is apparent from the Annual Reports of RASFF, e.g., the Preliminary Annual Report 2016 (46) that the number of notifications continues to increase in all notification categories, including alerts, border rejections, information for attention, and information for follow-up. Notifications expanded by 52% between 2006 and 2016, with substantial (17%) increase in border rejections, possibly due to Regulation (EC) No 669/2009 (47) imposing stronger border controls on food of non-animal origin, with systematic checks on documents accompanying all (100%) consignments, and routine physical checks, including laboratory analysis, at a frequency related to the risk identified. Certain notifications may correspond to the same sample, if multiple contaminants were above the official threshold of notification or intervention.

### Rate of Occurrence of VDs and PPPs as Contaminants in Europe

The four most prevailing causes of notifications in RASFF, representing over half of all notifications are mycotoxins, pathogenic microorganisms, pesticide residues, and heavy metals. Other causes are related to processing or treatment (e.g., foreign materials, non-pathogenic microorganisms, improper storage conditions, deviations in flavor and odor, and poor packaging) or deviations from legal requirements (e.g., improper composition, lacking documentation, non-declared allergen content, and erroneous labeling).

**Figure 1:**
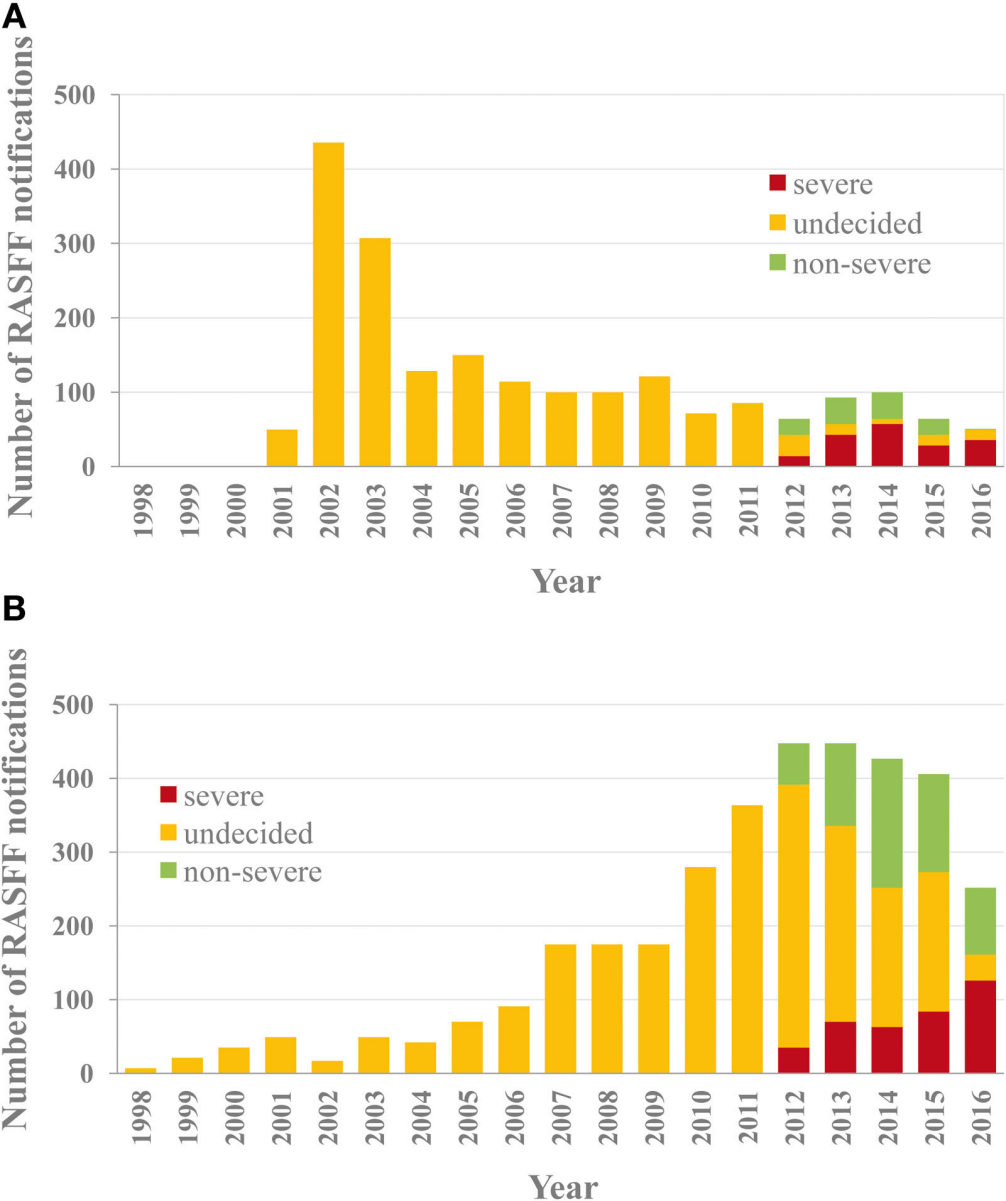
Notifications regarding residues of veterinary drugs **(A)** and pesticide residues **(B)** in agricultural products between 1998 and 2016 in the Rapid Alert System for Food and Feed (RASFF) database. The proportion of assessment-based risk severity decision categories is indicated after 2012.

A comparative analysis of violations found in RASFF for VDs and PPPs is rather informative. The overall numbers of RASFF notifications regarding VD and PPP residues between 2002 and 2016 were 2,036 and 3,527, respectively, indicating not only a 72% higher occurrence rate for pesticide residues but also different temporal trends. VD residues are a group of contaminants of lesser importance than the four groups mentioned earlier, as residues of pharmaceuticals (human and veterinary combined) represent only 4% of all notifications and are ranked 7^th^ among the causes of notifications. This relative ranking remained unchanged in the period of 2012–2016 (behind pathogenic microorganisms, mycotoxins, pesticide residues, heavy metals, additives, and contaminant migration), 46, 19, and 35% of which were severe, undecided, and non-severe cases, respectively. While the initial high number of reported cases in 2002 for residues of VDs has successfully been pushed to a level below 100 cases annually (Figure 1A), the number of reported violation cases for pesticide residues occurred to display a gradual increase from a low (approximately 50 cases annually) initial level after 2007–2010, and this tendency has come to a visible halt only by 2016 (Figure 1B). The opposing tendencies between the two sectors may be explained by their differing toxicology background: the toxicological requirements that apply for residues of human pharmaceuticals often provide substantial basis also for the assessment of VD residues, while such considerations are less expressed for pesticide residues. Toxicological rigor could effectively limit improper practices through firmness and proportionality of the measures taken in the regulation of veterinary medicine, unlike in the sector of pesticide residues. The difference became even more visible after 2012, when monitored data became subject to additional RA in RASFF. While the proportion of the category of “uncertain severity” decreased below 20% shortly after the introduction of the additional RA in RASFF (Figure 1A), it lengthily remained at 50% for pesticide residues, and this persisting tendency could be reversed only by 2016 (Figure 1B), also seen in the number of the documented cases. Uncertainty in decision-making can obviously not suppress improper practices effectively, as it cannot give ground to proportional measures taken.

The residues of the persistent active ingredients used in veterinary medicine repeatedly reach susceptible environments and habitats. It is quite common that pharmaceuticals already considered improper for humans still remain in use for a while as VDs and, in turn, still can reach the human body via food products of animal origin. Such cases were seen in the eighties for chlorinated hydrocarbons (not indicated by RASFF) and also lately for antimicrobials, the latter having been of growing concern regarding antimicrobial resistance appearing as a response to increasing chemical pressure on the environment due to antimicrobial VD residues (48, 49), particularly as antimicrobial resistance is known to emerge due to various environmental drivers (50) that should be a key policy aspect for environmental regulators. Numerous violation cases were recorded in RASFF in 2002–2003, when extensive monitoring was launched, and these cases were mainly related to crustaceans and other marine animals from aquacultures of Southern and Southeast Asia, and the safety status of the derived food products could be normalized only by 2010. The same can be said about apiculture products: the ban of honey import from China to the European market in 2009 resulted in a significant improvement in food safety. Similar spectacular advancements took place among poultry and fish products in 2004 and 2008, respectively. A different trend occurs, however, for mammalian farm animals, where numerous problems remain to occur in food production (e.g., pig, beef, and horse meat).

**Figure 2:**
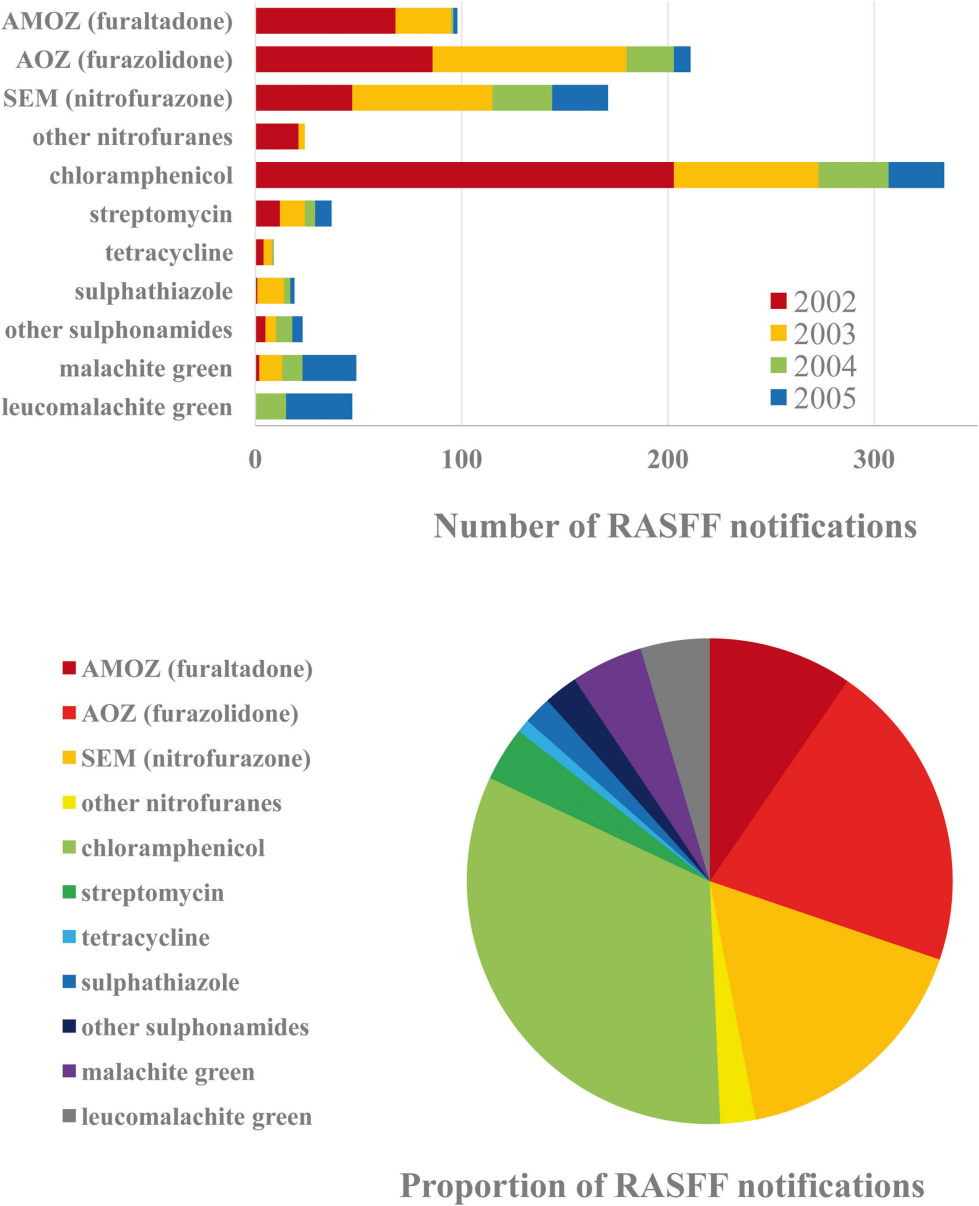
Frequently reported active ingredients of veterinary drug (VD) residues in the Rapid Alert System for Food and Feed (RASFF) database in the corresponding critical period, 2002–2005 (above). Proportions of the VDs reported during the 4-year period (below).

A detailed analysis of VD residues is most expedient to be carried out for the 2002–2005 period (Figure 2), when the largest number of notifications was issued. The corresponding period for pesticide residues is 2011–2015. Major countries of origin that have been identified as leading sources of notifications during the entire period of RASFF are Vietnam, India, and China, followed by Brazil, Thailand, and Bangladesh. As seen, countries of Southern and Southeast Asia are most frequently associated with questionable safety of food products of animal origin. Leading EU MSs as sources identified for VD residues in food products are Belgium, Poland, and Lithuania. It has to be mentioned, however, that these countries may be identified as contamination sources as importers.

Analysis of the 2002–2005 and 2012–2015 periods allow different conclusions regarding VDs. More than a decade ago, the overall incidence of nitrofuran type antibiotics was the highest in food products of animal origin, but individual occurrence of the dichloroacetic acid derivative chloramphenicol was the highest, and aminoglycoside streptomycin also occurred, although with substantially lower incidence, among notifications, moreover, several sulfonamides were detected. The most common within this last chemical type has been sulfathiazole, commonly used, in spite of its ban, in apiculture in China. Finally, malachite green (along with its metabolite in animals, leucomalachite green) and crystal violet (also known as gentian violet, previously used in aquaculture) can also be mentioned as contaminants detected.

Nitrofuran antibiotics are of outstanding significance among VD residues. Furaltadone [and its metabolite, 3-amino-5-morpholinomethyl-2-oxazolidone (AMOZ)] frequently occurred in poultry meat (e.g., chicken and sometimes turkey) from Brazil and occasionally from Thailand. The occurrence of furazolidone [and its metabolite, 3-amino-2-oxazolidinone (AOZ)] has been high in crustacean and fish shipments from South Asia, in egg products from India, pig and rabbit products from China, chicken from Thailand, and honey from Argentina. Residues of nitrofurazone [and its metabolite semicarbazide (SEM)] were common in freshwater shrimp from South Asia, lyophilized egg powder from Brazil, India, and France, chicken from Brazil and Thailand, and pig and rabbit from China.

Among antibiotics, chloramphenicol is known to widely occur in apiculture products. In addition, it has been detected in dairy products and commonly occurred in crustaceans and fish. Moreover, it has been detected in rabbit and duck meat and pork from China and duck meat from Thailand, as well as in duck and goose feed in Germany, which may explain the current situation.

Several changes have taken place by 2012–2015. The detection rate of AMOZ has decreased to a minimal level, and the statistics of AOZ occurrence has also improved, although the latter compound remains to occur in shrimp from Asia (India, China, and Malaysia) and rabbit from China. As a new emergence, it occurred in calf meat and also in animal feed above MRL in the Netherlands. The occurrence of SEM also shows a more favorable pattern by now, but as a new feature, it appeared in beef from Brazil and is a common contaminant of pangasius fish from Vietnam. As a result, the reputation of this fish, very well tolerating dense rearing conditions and only slightly sensitive to water contaminants, is rather unfavorable. The improvements are significant for the residues of chloramphenicol as well. Its incidence in apiculture products has dropped to casual occurrence after honey from China has been forced out from the EU market. The same applies to other antibiotics as well, indicating that one of the greatest successes in European food safety has been the regulation of apiculture products. Nonetheless, occurrence of chloramphenicol remains detectable in shrimp from China and Vietnam, as well as pork from China. Moreover, as it is still found in feed components from Belgium, France, and India, its casual occurrence has been indicated in various meat samples.

It has to be noted that in spite of the severe restrictions in the use of antimicrobials, the annual sales (and in turn, the anticipated usage) of these drugs remain high in Europe, particularly in Spain, Cyprus, and Italy, as reported by EMA in 2014 (51), and differences among countries in the use of antibiotics can be explained by different national regulations, prices, climate conditions, and animal demographics, as well as dosage regimes and the veterinarians’ prescribing habits. Among non-steroid anti-inflammatory drugs, residues of phenylbutazone in horse meat used for the treatment of the common degenerative disorder, chronic arthritis in horses, emerged as a new problem. The use of phenylbutazone has been substantially limited in the United Kingdom (UK), and it is currently registered for the treatment of race horses only. However, it can strongly be anticipated that this food safety problem, used to remain hidden due to the lack of control, existed before as well.

Feed additives are listed in a separate database within RASFF. The few cases detected (55 cases between 2012 and 2016) were limited to the poultry industry and mostly to residues of clopidol (48 of the 55 cases) used against coccidiosis and no longer permitted in the EU. No growth promoters are listed among the contamination cases found, which hints to the possibility that specific monitoring of these substances may not be sufficiently effective. It is well known that weight gain in cattle is promoted in the USA by the use of beta-blockers (e.g., ractopamine) that being one of the neuralgic points of the currently on-going Trans-Atlantic Trade and Investment Partnership negotiations. The use of the two best-known non-hormonal veterinary growth promoter preparations Zilmax (zilpaterol—Merck & Co.) and Optaflexx (ractopamine—Eli Lilly Co.) is not approved for animal husbandry in the EU, and ractopamine has been found in a horse meat sample from Mexico, as well as in beef liver from Canada according the RASFF database. Zilpaterol has been detected in horse meat from Mexico and surprisingly in poultry from Poland. In turn, wide scale monitoring of animal feed appears to be a problem that needs to be solved, as it would serve as an excellent prevention measure of contaminant dispersion.

**Figure 3:**
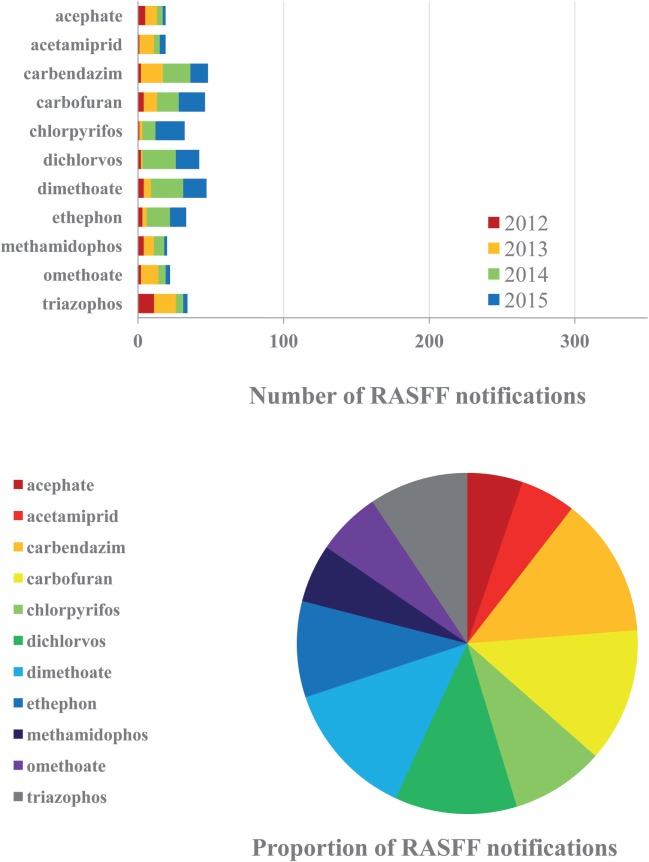
Frequently reported active ingredients of plant protection product (PPP) residues in the Rapid Alert System for Food and Feed (RASFF) database in the corresponding critical period, 2012–2015 (above). Proportions of the PPPs reported during the 4-year period (below).

The most complex issue in the RASFF database from the aspect of analytical determination and assessment is unquestionably represented by pesticide residues. Initial findings indicated severe warning signs as early as in 2002, immediately after the launch of the operation of RASFF, yet pesticide residue levels remained to display a trend of continuous increase until recently. This segment with over 75 severe cases as an average annually on the basis of the last five years (2012–2016) is likely to be considerably underestimated among food safety hazards. The majority of the findings have been related to pesticide active ingredients not enrolled on the EU positive list of registered compounds. Related PPPs, however, may be legally used in exporting non-EU countries, and therefore, their residues may be found in feeds or in foods of animal origin produced there. In such cases, shipments with any detectable amounts of the given residue are rejected, even if the level remains below the earlier MRL. The other large proportion among RASFF findings correspond to the occurrence of residues of pesticide active ingredients registered in the EU, above the corresponding MRLs. Approximately two-thirds of pesticide residues reported by RASFF between 2012 and 2016 belonged to the first group, i.e., disapproved shipments were contaminated with residues from technologies no longer applicable within the EU, and only one-third of the reported pesticide residues belonged to active ingredients authorized in the EU. Moreover, the proportion of RASFF notifications among the target analytes specified appears to be quite even. The most severe current cases of residues of banned pesticide active ingredients include carbendazim (fungicide), carbofuran, dichlorvos (zoocides), and ethephon (ripening accelerator), as well as still authorized active ingredients dimethoate and chlorpyrifos (zoocides) (Figure 3). A recent, severe, but isolated issue has been the case of insecticide fipronil found in eggs and egg products in 2017. Fipronil is used both in VDs and PPPs. Its veterinary use is against fleas, mites, and ticks mostly on dogs and cats, e.g., in formulated VD products Frontline, Fiproguard, Flevox, Petarmor, and Sergeant, but Frontline has been approved for poultry, for bird and housing treatments for external parasites as well, and possible emergence of fipronil residues in eggs is known since 2001 (52). In PPPs, it is used against a wide range of insect pests. After gradual limitations of its use (e.g., strictening the use of its formulated product Regent in Hungary in 2008), fipronil was banned in the EU in 2013 from use on animals destined to enter the food chain. Over the years, residues of this insecticide have been found in commodities of plant origin (notified in most cases as border rejection), yet it was found in eggs from Belgium in 2017 at concentrations up to 1.2 mg/kg (notified as an alert of serious risk), indicating illegal use of this substance in the poultry sector and possible human health risk from contaminated eggs.

### Network Analysis of the Non-Compliance Cases Reported in the EU RASFF

**Figure 4:**
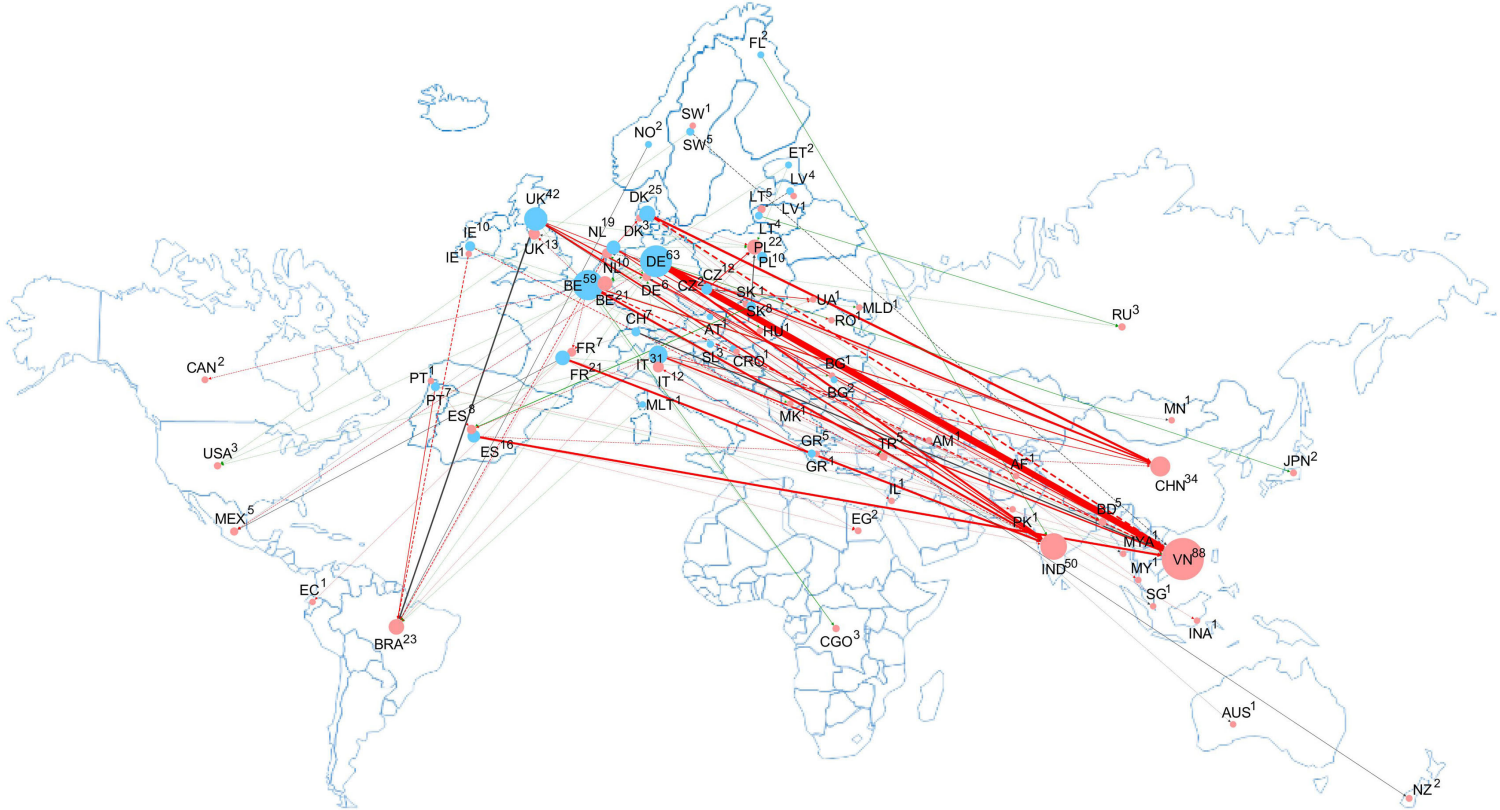
Connection network among notifier and consigner countries in the Rapid Alert System for Food and Feed database regarding food/feed contamination with veterinary drug residues between 2012 and 2016. Notifier and consigner countries are designated with blue and red circles, respectively, with the number of reported cases indicated near the country code and circle sizes proportional with numbers of reported cases. Thicknesses of the connecting lines (dashed line for single and solid line for multiple case notifications) are proportional with overall notification cases in the given relation, and colors of the connecting lines corresponding to the risk assessment category of the contamination cases found (red: severe cases were identified; gray: no severe cases, but cases of undecided severity were identified; and green: solely non-severe cases were identified). (Note that Europe is shown larger than proportional on the background world map for better connectivity visibility.)

Mapping non-compliance cases and alerts in RASFF regarding VD residues in food and feed among EU countries and food/feed supplier countries is an informative tool in identifying the sources of non-compliances on the EU markets, if the consigner country of the notification is indeed the country of origin. It has to be noted, however, that contamination is not always detected immediately at source, and in such cases, the consigner country is an importer that further exports the commodity reported in RASFF. Claims may be (and are mostly) related to products originated from outside the EU. Figure 4 summarizes and illustrates RASFF notifications on VD residues in food and feed in the EU in the period when notifications are the most informative, supplemented by RA categorization (between 2012 and 2016). The network of the notification cases not only illustrate the actual relations of complaints but also provide a more accurate picture of the control system within the EU. The network map shows that most non-compliance cases were identified in relation to Vietnam and the main notifiers were Germany and Belgium.

Within the 5-year period between 2012 and 2016, there occurred 362 notifications, 67 (nearly one-fifth) of which were domestic notifications (with the notifier and consigner country being the same), indicating either domestic production or unidentified import. With this value, residues of veterinary pharmaceuticals ranked 7^th^ (among all notifications, 168 cases, 46% of all cases were assessed as severe). Consigning countries of extensive non-compliances included Vietnam, India, China, and Brazil (88, 50, 34, and 23 notifications, respectively). Vietnam scores particularly poor in the notifications regarding VD residues, as otherwise the country is ranked at a much better, 14^th^ position in the overall RASFF notifications from 1998 until the first quarter of 2017 (1,296 notifications). As for the other three countries, China, India, and Brazil are ranked 1^st^, 3^rd^, and 12^th^ in the overall RASFF notifications (nearly 5,540, 2,966, and 1,618 notifications, respectively). The relative ranks of the overall notifications for these four countries remained unchanged also regarding the complaints received between 2012 and 2016, and as for their RA, those assessed as corresponding to severe risk represented 30–55% for these countries. For VD residues, the overall severity rank increased from 2012 to 2014, but later displayed a favorable decreasing trend along with a parallel decrease in the number of all notifications. The network is dominated by a Germany—Vietnam axis (31 notifications, 11 of which were severe), along with strong notification connections also at other source countries mentioned above. The notifications toward Vietnam were assessed predominantly as severe by Spain, Italy, the Netherlands, and Switzerland and to a less degree by Germany, Belgium, and the UK. Predominantly severe notifications were reported toward India by Belgium, France, and the UK, with less severity from Germany. Thus, countries of South and Southeast Asia are considered a vulnerable point with regard to VD residues entering the EU market.

Although the RASFF documentation reports notifications only, and not the overall number of samples analyzed, it indicates that lead monitoring EU countries for all food and feed contaminants on the basis of their reported notifications are Italy (7,981 RASFF notifications from 1998 until the first quarter of 2017), followed by Germany, the UK, Spain, the Netherlands, France, Belgium, and Denmark (6,571, 5,130, 3,741, 3,180, 2,671, 1,782, and 1,540 notifications, respectively). These same countries were reporting the highest numbers of VD residues found between 2012 and 2016 but in a slightly different order: Germany, Belgium, the UK, Italy, Denmark, France, the Netherlands, and Spain (63, 59, 42, 31, 25, 21, 19, and 16 notifications, respectively). The numbers of notification cases in the official monitoring in each country indicate that not only the operation of the food safety sector at the European level is a determining factor, but the national food safety organizations, of which the Federal Institute of Risk Assessment (BfR) in Germany is of outstanding weight, also represent an equally important contribution. It has also to be noted the non-EU countries, particularly Norway and Switzerland, also provide data to the RASFF database.

### The Range of Target Analytes in the EU RASFF

Rapid Alert System for Food and Feed monitors food/feed contaminants according to its legal mandate: its target analytes include pathogenic and non-pathogenic microorganisms, mycotoxins, PPP, and VD residues, allergens, foreign materials, industrial and biocontaminants, food and feed additives, as well as improper compositions, genetically modified components or adulteration. These contaminants, covered within the RASFF activities are regulated by legal MRLs, threshold levels or critical content for mandatory labeling. The MRLs specified, e.g., in Regulation (EC) 470/2009 (10) apply only to pharmacologically active components but not to “inert” substances. In turn, RASFF does not cover excipients, because these components are—often erroneously—considered “inert” substances. They are, indeed, inert *per definitionem* in the main effect of the formulation they are used in, but they may also exert adverse side effects. Emerging information on the hazards of risks related to formulant additives indicates that some of these excipients should be included among target analytes in RASFF; in other words, MRLs should be defined for these substances as well. The EU-wide regulation of adjuvants and co-formulants is being planned; however, their monitoring is hindered by the facts that analytical methods for their determination are often missing, and quantitative analysis is often problematic for these complex, in given cases not fully described substances. Moreover, the effect of these excipients on the residue levels recorded for the active ingredient is hardly studied.

## Excipients, Additives, and Adjuvants

Beside the active ingredients, several additives can also be found in formulated animal therapeutic agents and feed additive products, as well as in the formulated pesticide preparations. Among additives, classified into several groups by their function, adjuvants are a minor group of substances, used for the primary purpose to enhance the biological effect of the active ingredient (13, 53). Thus, adjuvants (e.g., various surfactants, solvents, dispersing agents, activators, wetting or antifoaming agents, anti-evaporants, drift retardants, softeners, safeners, stabilizers, and penetrants) directly affect the efficiency of the formulations. Further groups of additives are not used for the purpose of amending formulation efficiency but implement other purposes related to application, such as the promotion of safe use and application ensured by colorants and odorants (54). For example, the warning effect of the red dye used to be applied in carbofuran-based formulations or the unpleasant smell of odorants applied in obsoleted formulations containing paraquat or diquat used to serve the purpose of lowering the possibility of human poisoning during use and application of the formulations (55, 56). Additionally, other groups of additives consist of various trapping agents and attractants, which also do not affect directly the efficiency of the active ingredient (13, 57, 58). As seen from the above, the often seen practice of using additives and adjuvants as synonymous words is incorrect.

### Surfactants

A characteristic feature in the chemical structure of different surfactants is the simultaneous presence of hydrophobic and hydrophilic moieties; therefore, surfactants show both lipophilic and hydrophilic properties (59, 60). The estimated annual world production of surfactants was at 15 million tons in 2005 (61). Besides the industrial (e.g., laundry detergents and cleaning supplies, detergents in cosmetics, and engine oil additives) and domestic (e.g., domestic laundry and dishwashing detergents and soaps) application of various surfactants (summarized in Table 1), the use in VDs and PPPs represents a substantial sector, as well. Surfactants enhance the efficiency of formulations by increasing the water solubility, bioavailability and biological activity of the active ingredients (62, 63). Surfactants may be used to solubilize drugs through micellar dispersion in VDs (64), furthermore, are applied in feed additives applied in drinking water as stabilizers to prevent decomposition of the active ingredient(s) in the preparation (65). Various types of surfactants used in veterinary medicine and in feed additives are summarized in Tables 2 and 3, respectively. In addition, surfactants or wetting agents enhance drug solubility and membrane permeability, prolong gastrointestinal residence time, and protect the active ingredient from luminal degradation and metabolism in the gut wall (66). Enhancement of bioavailability of polar compounds without affecting solubility characteristics can be achieved by absorption enhancers (e.g., anionic and non-ionic surfactants, acylamino acids, acylcarnitines, and lysolecithin) (67–69). Conversely, surfactants also applied to increase the *in vitro* solubility of lipophilic compounds (70, 71). Formulation is of particular importance for PPPs, as additives may aim not only to improve the solubility, adsorption, or penetration of the active ingredient in these formulations but also to enhance environmental stability, bioavailability, and capability to reach the site of action. Various types of surfactants used in pesticide formulations are summarized in Table 4. Surfactants are generally classified according to the type of their hydrophilic part; therefore, anionic, cationic, non-ionic, and amphoteric surfactants can be distinguished (72).

**Table 1:** Various types of surfactants used for general purpose.

Chemical name	Product name	Type	Producer/supplier	*CAS* number
Sodium dodecyl benzene sulfonate	Neopelex G-65	Anionic	Kao Chemicals	25155-30-0
Lauryl glucoside, sodium lauryl glucose carboxylate	Plantapon LGC	Anionic	The Soap Kitchen	383178-66-3, 110615-47-9
Sodium xylene sulfonate	Stepanate SXS-93	Anionic	Stepan	1300-72-7
Cetyl trimethyl ammonium chloride	Dehyquart A-CA	Cationic	BASF	112-02-7
Lauryl dimethyl betaine (quaternary ammonium compound)	Emulson AG CB 30	Amphoteric	Lamberti SpA	66455-29-6
*n*-Dodecyl-*n*,*n*-dimethyl-3-ammonio-1-propanesulfonate	Zwittergent 3-12	Amphoteric	Merck Millipore	14933-08-5
Alkyl polyglucoside (lauryl glucoside)	Kemgluko CLM	Non-ionic	KemCare	110615-47-9
Cocamide diethanolamine	Amidet B-112	Non-ionic	Kao Chemicals	68603-42-9
Octylphenol ethoxylate	Triton X-100	Non-ionic	Dow	9002-93-1

**Table 2:** Various types of surfactants used in veterinary drugs or disinfectants.

Chemical name	Product name	Type	Producer/supplier	*CAS* number
Dioctyl sodium sulfosuccinate	Vedco Veterinary Surfactant	Anionic	Respa Pharmaceuticals Inc	577-11-7
Didecyl dimethyl ammonium bromide	Bromosept 50	Cationic	ABIC Biological Laboratories Teva Ltd	2390-68-3
Alkyl dimethyl benzyl ammonium chloride (C_12–18_) (ADBAC)	Dec-quat 100	Cationic	Veltek Associates Inc	68391-01-5
Alkyl dimethyl ethyl benzyl ammonium chloride (C_12–14_) (ADBAC)				85409-23-0
Polyethylene glycol (PEG) glyceryl stearate	Gelucire 50/13 Gelucire 50/02	Non-ionic	Gattefossé SAS	9011-21-6
PEG glyceryl laurate	Gelucire 44/14	Non-ionic	Gattefossé SAS	57107-95-6
PEG-8 caprylic/capric glycerides	Labrasol	Non-ionic	Gattefossé SAS	61791-29-5
12-Hydroxystearic acid-polyethylene glycol copolymer	Solutol HS 15	Non-ionic	BASF	70142-34-6
Sorbitane ester ethoxylate	Polysorbate 80	Non-ionic	Croda Americas, Inc.	9005-65-6

**Table 3:** Various types of surfactants used in feed additives.

Chemical name	Product name	Type	Producer/supplier	*CAS* number
Sodium lignosulfonate	Arbo S01P	Anionic	KemTek Industries Inc	8061-51-6
	Borresperse Na		Borregard Ligno Tech	
Calcium lignosulfonate	Borresperse Ca	Anionic	Borregard Ligno Tech	8061 52 7
Linear calcium dodecylbenzene sulfonate	Rhodacal 60/BE	Anionic	Solvay & Rhodia	26264-06-2
Glycerol-polyethylene glycol ricinoleate	Volamel Extra	Non-ionic	Nukamel	61791-12-6
	Alkamuls SC/242		Solvay & Rhodia	
Alcohols, C_8–10_, ethoxylated propoxylated	Antarox BL 225	Non-ionic	Solvay & Rhodia	68603-25-8

**Table 4:** Various types of surfactants used in plant protection products.

Chemical name	Product name	Type	Producer/supplier	*CAS* number
Alkyl (C_8–10_)-polyoxyethylene ether phosphate	Rolfen Bio	Anionic	Lamberti SpA	68130-47-2
POE alkyl phosphate ester				50769-39-6
Dioctyl sulfosuccinate sodium salt	Imbirol OT/NA/70	Anionic	Lamberti SpA	577-11-7
Sodium-alkyl polyglucoside citrate	Eucarol AGE-EC	Anionic	Lamberti SpA	151911-51-2
Sodium-alkyl polyglucoside sulfosuccinate (in aqueous solution)	Eucarol AGE 91/S K	Anionic	Lamberti SpA	151911-53-5
Sodium dodecyl benzene sulfonate	Agrosurf WP85	Anionic	Lankem Ltd	25155-30-0
Secondary alcohol ethoxylate	Tergitol 15-S-9	Non-ionic	Dow Chemicals	68131-40-8
POE (15) tallow amine formulated	Emulson AG GPE3/SSM	Non-ionic	Lamberti SpA	61791-26-2
Non-ylphenol polyethylene glycol ether	Triton N-57	Non-ionic	Dow	127087-87-0

#### Anionic Surfactants

Various anionic surfactants, containing functional groups capable to dissociate to form anions as the polar part of the molecule [e.g., carbonates, sulfates, and most of all sulfonates, such as linear alkylbenzene sulfonates (LASs) and alkyl sulfonates], are frequently used in large quantities in VDs, feed additives, and PPPs. Anionic surfactants can enhance the biological efficacy of the active ingredient (73, 74) through direct binding to it (75) or modification of its adsorption. Moreover, they can act as enzyme activators or inhibitors by binding to the enzyme protein in a concentration-dependent manner and their binding affinity depends on the length of the alkyl chain in the surfactant (76). LASs can inhibit alkaline phosphatase and acid phosphatase enzymes (77), and sodium dodecyl sulfate (SDS) improves the intestinal absorption of active ingredients, e.g., the anthelmintic drug albendazole (78). Further surfactants, e.g., calcium dodecylbenzene sulfonate and lignosulfonate (e.g., Arbo), are used for the formulation of feed additives and PPPs. Perfluorinated sulfonates and carboxylic acids, including perfluorooctanoic acid and perfluorooctane sulfonate—suspected environmental endocrine disruptors—have been in use for over 50 years (79). Beyond agrochemical applications, the industrial use of several anionic surfactants, such as calcium dodecylbenzene sulfonate (Rhodacal 60/BE), sodium dodecylbenzene sulfonate (Neopelex G-65), ammonium lauryl sulfate (ALS), and sodium lauryl sulfate, in the formulations of laundry detergents and cleaning supplies is also significant (58, 72, 80). Sulfonates are among the most widely used anionic surfactants in personal care and household products (81, 82).

#### Cationic Surfactants

The polar part of cationic surfactants contains cation-forming functional groups. Among these, the representatives of primarily use are quaternary ammonium compounds (QACs), applied as disinfectants and cleaners, due to their advantageous adsorptive and bactericidal properties, in agricultural practice and veterinary medicine (83). The most commonly used QACs in veterinary and animal health practice are benzalkonium chloride (Bradophen), dialkyl dimethyl ammonium chlorides (ADBACs), and the so-called fourth generation of QACs, e.g., dioctyl dimethyl ammonium bromide and didecyl dimethyl ammonium bromide.

#### Non-Ionic Surfactants

In the molecular structure of non-ionic surfactants, a polyethylene glycol (PEG) moiety is connected to alkylphenols [i.e., alkylphenol ethoxylates (APEs), e.g., octylphenol (OP) and nonylphenol (NP) ethoxylates, suspected to exert hormone modulant effects; or long chain fatty alcohols, acids, or amines, e.g., alkylamine ethoxylates (ANEOs), polyethoxylated tallow amines (POEAs), fatty alcohol ethoxylates (AEOs), and fatty acid ethoxylates]. OP and NP derivatives are generally used in the production of non-ionic APEs (58, 84). In enterosolvent capsules used in veterinary medicine, water-miscible non-volatile and non-ionic surfactants are used for formulating poorly water-soluble compounds (85). Moreover, non-ionic surfactants are generally used as emulsifying or dispersing agents, emulsion stabilizers and binders in VDs, and feed additives (64). Non-ionic surfactants are generally applied as detergents in the industry and as formulating agents in PPPs (80). Additives for industrial use, such as cocamide monoethanolamine and diethanolamine (DEA), are used as foaming agents in different soaps, shampoos, and cosmetics, but despite their advantageous characteristics for industrial purposes, cocamide DEA has been classified to category 2B, possible human carcinogen, by the International Agency for Research on Cancer (86). Alkyl polyglycosides (APGs), glyceryl laurate (e.g., monolaurin), and glycerol-polyethylene glycol ricinoleate (Volamel Extra) are often used as feed additives (e.g., emulsifier and stabilizer), due to their effect of increasing the digestibility of the animal feed (87). Polyethermethylsiloxanes, as trisiloxane surfactants, are often used in pesticide formulations to enhance the activity, efficiency, and the rain fastness of the active ingredient, due to their hydrophobic properties (88). Other surfactants for formulating PPPs include sodium alkylpolyglucoside citrate (Eucarol AGE-EC), POEA (Emulson AG GPE 3SS), and secondary AEOs (Tergitol 15-S-9) (89). A particular feature of OP ethoxylate (Triton X-100), as a non-ionic surfactant, is its capability for the lysis of integral membrane proteins; therefore, Triton X-100 is substantially used in biochemical studies (90, 91). Non-ionic surfactants are considered to exert lower toxicity than cationic, anionic, and amphoteric surfactants (59, 60). APGs are called “green surfactants” due to their low environmental impacts (92). However, the toxicity profile of tallow derivatives (e.g., POEA and hydrogenated tallow glycerides), used as surfactants in VDs and in PPPs as well, has recently become of significant importance in (eco)toxicological assessment (see “Tallow Derivatives” below).

#### Amphoteric Surfactants

Due to their zwitterionic structure, e.g., showing anionic and cationic characteristics simultaneously, amphoteric surfactants have high water solubility and show low contact toxicity characteristics, e.g., favorable dermatological and low eye irritation properties. In turn, amphoteric surfactants gained extensive use in cosmetics but are also widely used as adjuvants in agrochemicals. Their main groups are betaines, sultaines, iminodiacids, and acyl ethylene diamines (58, 80).

#### Biosurfactants

Natural surface-active substances are produced by plants, animals, and microorganisms (93). These biosurfactants, such as monoacylglycerols and their derivatives (e.g., ethoxylated monoglycerides, acetic, and diacetyl tartaric esters of monoglycerides) obtained from animal and plant lipids, including beef tallow, as well as rapeseed, lard, olive, and palm oils are widely used as emulsifiers in cosmetics, pharmaceutical industries, and foods (94–96). Additional biosurfactants used in veterinary preparations include wax and fat compounds (e.g., hydrogenated tallow, triglycerides, PEGs, fatty alcohols, fatty acids, or stearates) (64). Several various anionic and neutral biosurfactants are known, but cationic biosurfactants have been described extremely rarely, probably due to their toxic effect (97). Generally, biosurfactants are considered biodegradable and relatively non-toxic (93). Biosurfactants, such as surface-active sophorolipids, assure surface-lowering properties, advantageous biodegradability, and low ecotoxicology, and are used in cosmetics, pharmaceuticals, and medical preparations due to their biological effects and activity (98).

#### Tallow Derivatives

Generated wastes by the oil and fat industries, such as residual oils, lard, and tallow, are additional sources of cationic biosurfactants for fabric softeners. In addition, non-ionic tallow derivatives are used as surfactants in VDs and PPPs (99, 100). These substances are manufactured from biological resources via industrial chemical synthetic processes, therefore, are considered industrial chemicals. As seen above, surfactants derived from animal tallow, as non-ionic substances, have wide application in formulation of both veterinary products and PPPs. Yet, the biological origin cannot be considered as a guarantee for favorable toxicological characteristics, as indicated by several examples. Food, feed, and environmental safety of tallow have been assessed by EFSA (101) and EMA (102) only with regard to transmissible spongiform encephalopathy (TSE) infectivity. Despite possible TSE risk connected to tallow is considered by the Scientific Steering Committee of the EC, originated from protein impurities may be present in the final products (103), the EFSA scientific opinion document states that in general, the risk can be regarded as minimal on the basis of the calculated levels of exposure evaluated by quantitative risk analysis. The conditions of the application of concerning animal by-products (e.g., tallow used as raw material for manufacturing tallow derivatives) are governed by Regulation (EC) No 1774/2002 (104). Upon being separated from animal fat via heat treatment (e.g., “fat melting”), moisture content reduction, and lipid separation, tallow is often subjected to chemical derivatization and corresponding tallow derivatives are occasionally also far from being unproblematic in their toxicity features, in spite of their long being considered as “inert ingredients” or “inert additives.” The high toxicity of POEA, related to ANEOs, used primarily as a non-ionic formulating agent in glyphosate-based herbicides, was proven by several studies (105–108). POEA consists of a tallow amine moiety and two chains of repeating ethoxylate units. The tallow amine moiety is a mixture of amines derived from palmitic acid, stearic acid, oleic acid and other minor components (73). Non-ionic hydrogenated tallow glycerides are used as dispersing agents, emulsifying agents, emulsion stabilizers, and binders in VDs (64). Similarly, polyethoxylated mono- and diglycerides of tallow fatty acids are also listed in the corresponding EU lists of authorized substances.

### Surfactant Usage in VDs and PPPs

**Figure 5:**
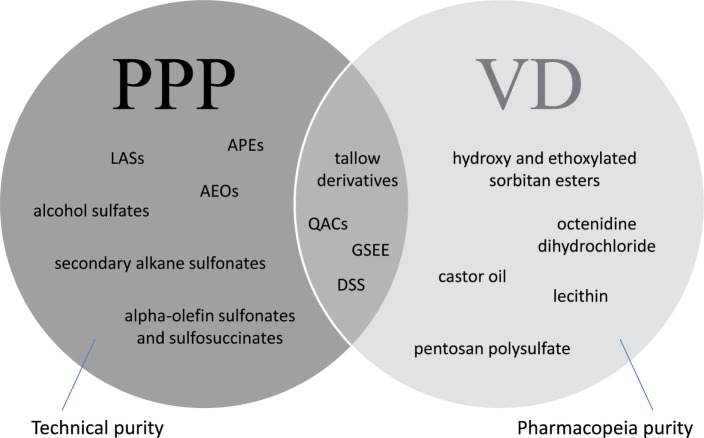
Surfactants used in veterinary drugs (VDs) and plant protection products (PPPs) indicating exemplary substances used particularly in VDs, in PPPs (APEs, alkylphenol ethoxylates; LASs, linear alkylbenzene sulfonates; AEOs, alcohol ethoxylates), or in both groups (tallow derivatives: hydrogenated and polyethoxylated tallow substances, QACs, quaternary ammonium compounds; GSEEs, glycerol sorbitane ester ethoxylates; DSS, dioctyl sodium sulfosuccinate). Additives in VDs are required to be of Pharmacopeia purity, while formulants in PPPs can be used in technical purity.

Surfactants used in formulated VDs and PPPs may be characteristic to one or both of these product groups (Figure 5). Thus, certain substances, e.g., sorbitan esters and their ethoxylated derivatives, octenidine dihydrochloride, castor oil, pentosan polysulfate or lecithin are being used as excipients for VDs, but not for PPPs, while other compounds, e.g., APEs, LASs, AEOs, and alpha-olefin sulfonates and sulfosuccinates, are typically used for the formulation of PPPs. Certain substances, e.g., hydrogenated or polyethoxylated tallow derivatives, QACs or glycerol sorbitane ester ethoxylates, and alkyl sulfosuccinate salts, e.g., dioctyl sodium sulfosuccinate, may be used both for VDs and PPPs; however, it has to be emphasized that their chemical moiety is not equivalent even in these cases, as additives in VDs have to meet Pharmacopeia purity requirement, while regulations of PPPs allow the use of these additives in technical purity.

In 1998, the estimated global use of major classes of surfactants was 1.77, 0.35, 0.32, and 0.30 million tons for LASs, AEOs, APEs, and alcohol sulfates (109). Moreover, the annual global production of synthetic surfactants was about 7.2 million tons (59) and 2.8 million tons for the most popular synthetic anionic LASs (110). In the USA alone, the quantity of produced surfactants was at 3.5 million tons in 1999 and 35% of these were bio-based (111). In 2000, the total consumption of secondary alkane sulfonates was at about 0.072 million tons in Western Europe, while the application of alpha-olefin sulfonates and sulfosuccinates were 0.006 and 0.009 million tons, respectively (112), while annual usage of detergents and softener products were 4.25 and 1.19 million tons, respectively (113). Unfortunately, no details are readily available regarding the proportion of surfactants used in VDs and PPPs within these global trade values, but practically all of the chemical classes mentioned earlier are represented in this segment as well with the corresponding registration requirements considered. Thus, consumable surfactants registered to be used in VDs include castor oil ethoxylates, sorbitan esters, and their ethoxylated derivatives, as well as lecithin. Nonetheless, substances of less uniform characteristics, e.g., hydrogenated and polyethoxylated tallow derivatives, can also be used. The reported global production of surfactants was 8.6 million tons in 2003 (114). In 2005, the estimated annual world production of surfactants was at 15 million tons (61). Production and global use of non-ionic surfactants are continuously growing (115). Anionic surfactants emerged as the largest segment of the surfactants market in 2014, responsible for more than 45% of the global market; moreover, the global market of surfactants reached 20.2 billion US$ (116, 117). In 2015, it was estimated to 30.65 billion US$ (118). The overall surfactant market has been showing a constant growth in the last years, with the USA, China, Western Europe, and Asia being responsible for the largest rate of surfactant consumption (119).

### Ecotoxicological Effects of Surfactants

Additives used as surfactants in VDs, feed additives, or PPP formulations may have adverse effects on the environment and on non-target organisms. The cytotoxicity order of surfactants investigated on rabbit corneal epithelial cells was found to be cationic > anionic = amphoteric > non-ionic (120). Surfactants may influence the embryonic development and hormonal balance of vertebrates, mainly in aquatic habitats, and genotoxic effects have been indicated for several types of surfactants (121–125). Lewis and Supernant investigated the effects of three types of surfactants, anionic C11.8 LAS, cationic cetyl trimethyl ammonium chloride (CTAC), and non-ionic C_14-15_ alkyl ethoxylates (AEOs), on several aquatic invertebrates and fish species. The order of the toxicity level was found to be AEO > CTAC > LAS (126). Singh and co-workers investigated the effects of several surfactants on fish species. The toxicity order of the investigated surfactants was cationic surfactants > anionic surfactants > non-ionic surfactants (127). Interestingly, the toxic effect of monoalkyl QAC surfactants was not proven to increase with the alkyl chain length in the molecules (128). Anionic LASs have been shown to be uptaken by fish from water via the gills rather than the skin. The concentration of LAS surfactants increases rapidly in the liver and other internal organs of fish juveniles (129). Bioaccumulation in the aquatic environment is higher than in the terrestrial environment in the case of LASs (130). Pavlic et al. investigated the effects of nine detergent ingredients on algae species. Non-ionic detergent (decyl polyglycoside) exerted higher toxicity than anionic (e.g., sodium lauryl ether sulfate and ALS) or amphoteric (alkylamidopropyl betaine and alkylamidoethyl-*N*-hydroxyethyl glycine) ones (131). Jurado and co-workers investigated the effects of three APGs of different polymerization rates and alkyl chains, and toxicity increased with the alkyl chain length (132). An opposite role of the alkyl chain length of AEOs in the acute toxicity on the water flea, *Daphnia magna*, has been found in several studies (133, 134). LAS detergents caused abnormalities in the development in several marine invertebrates (135). NPs and OPs, as biodegradation products of APEs, exert toxicity on freshwater and marine fish species (136) and induce estrogenic responses (137, 138). Given APEs, e.g., NP ethoxylate, are suspected environmental endocrine disruptors, exerting hormone modulant effects themselves or through their AP metabolite, mostly as estrogen agonists (139, 140) or androstane agonists (141). Thus, the estrogenic activity of APs was demonstrated both *in vitro* (142) and *in vivo* (143). At molecular level, APs are capable to bind to estrogen receptors in fish and mammals (144, 145) and to activate reporter genes regulating estrogen-responsive elements (146, 147). Moreover, in aquatic animals, APs are capable to interfere with steroid metabolism (148) and steroid hormone receptor activity (149). Antiandrogenic activity due to altering aromatase activity and impeding the function of aryl hydrocarbon receptors has also been detected (150). Moreover, possible enhancing effects of given active ingredients (e.g., atrazine) and NP on 7,12-dimethylbenz[a]anthracene-induced mammary tumor development in human c-Ha-ras proto-oncogene transgenic rats have been evidenced (151).

The toxic effect of additives in PPPs has been clearly demonstrated by several studies in which formulated pesticide products were proven to be more toxic than their active ingredient alone (106, 152). Recently, the investigation of the combined toxicity of the worldwide most used herbicide active ingredient glyphosate and surfactant POEA as its most common formulant received special attention, as scientific evidence indicated higher individual toxicity of the surfactant or combined synergistic effects between the active ingredient and surfactants. The effects of POEA and a glyphosate-based herbicide formulation (Roundup) on different test organism were compared by Chu and Tsui, and POEA proved to be more toxic (106). The acute toxicity of glyphosate, a glyphosate-based formulation, and the surfactant applied in given formulation on aquatic invertebrates and fish species were investigated by Folmar et al., and POEA was proven to be the most toxic component, compared to the effects of technical grade glyphosate and the investigated formulation (105). In a later study, ethoxylated adjuvants used in glyphosate-based formulations proved to be nearly ten thousand times more toxic than the toxicity of the active ingredient (107). This finding has been reconfirmed in numerous additional studies (108, 153); moreover, several studies verified POEA as the most toxic component on *D. magna* as well (108, 154). The permeability of cell membranes can be affected by POEA, resulting in the enhancement of the absorption capacity of the biologically active agents, their cytotoxicity and effects on the cells inducing apoptosis or necrosis (155). On the basis of these findings, POEA as a formulating agent was proposed to the MSs to be excluded from glyphosate-based pesticide formulations in the EU in 2016 (156). The ban includes numerous PPP formulations, including Roundup Classic, Roundup Classic Plus, Roundup Forte, as well as numerous other products under trade names other than Roundup.

### Combined Effects: Synergism, Additive Effect, and Antagonism

Interactions may occur between the active ingredients and additives used in formulated VDs, feed additives or pesticides. Due to their parallel presence in the given formulations, these substances may modify each other’s effects, and their combined effects may be additive, synergistic, or antagonistic (157). Combined toxicity of active ingredients has been confirmed recently in several studies (158); furthermore, the individual toxicity of several additives was verified as well (106, 152, 159). The simultaneous application and presence of non-ionic amine oxide-based surfactants and anionic surfactants in formulations has been proven to result in synergistic effects between the surfactants (160, 161).

As a consequence of the above mentioned results, the assumption that additives used in formulations are inactive (inert) ingredients has been falsified is numerous cases and should be considered significantly questionable on the basis of the scientific evidence. Combined effects of various active ingredients and surfactants have been confirmed in veterinary medicine as well. Antagonistic effects between various bacteriostatic and bactericidal compounds and synergistic effects between antiseptic anionic tensides and other disinfectants (e.g., hexachlorophene) have been observed. Moreover, the dissociation, α-chymotryptic degradation, and enteral absorption of insulin hexamers are influenced by the combination of SDS and the cationic cetyl trimethyl ammonium bromide surfactants in pharmaceuticals (162).

Combined toxicity and synergistic effects between active ingredient and formulating agents used in formulation of PPPs; moreover, the individual toxicity of surfactants applied in formulations were proven by several studies (108, 152, 153, 163). Various PPPs used in chemical plant protection were proven to be more toxic than the corresponding active ingredient, especially to aquatic organisms (108, 152). The toxicological evaluation of surfactants and other ingredients is essential for proper and effective ERA of formulations used in veterinary and agricultural practice.

### Environmental Fate of Surfactants

Little information is available regarding the environmental fate of adjuvants (e.g., surfactants) after the application in VDs and PPPs (72). As a result of the significant production and industrial, agricultural, and domestic use, surfactants, their metabolites, and decomposition products can easily enter into environmental matrices, including soil, sediment, surface water, and even drinking water (58, 164, 165). A significant source of pollution is chemical plant protection, and also inadequate or uncontrolled management and treatment of wastewater and sewage sludge. Among different groups of environmental endocrine disruptors, e.g., drinking water contaminants, pesticide residues, surfactants, and industrial pollutants are highlighted (166).

Surfactants may sorb directly onto the surface of the solid phase in soil and sediment, or may interact with sorbed surfactant molecules as well (167–169). The adsorption capacity of surfactants is highly dependent on their physico-chemical characteristics (170). Cationic surfactants adsorb strongly onto the particles of soil and sediment (171), and the order of adsorption rate and affinity of surfactants is cationic > non-ionic > anionic (60), with cationic and non-ionic surfactants showing much higher sorption on soil and sediment particles than anionic surfactants (e.g., LASs). The degradation of APEs is faster in water than in sediment (172), and their metabolites are degraded more easily under aerobic than under anaerobic conditions (112, 173). In contrast, fatty AEOs are equally degradable in aerobic and anaerobic environments (174). Most of the surfactants can be degraded by microorganisms; however, various surfactants, such as LAS, dehydrogenated tallow dimethyl ammonium chloride, and APG, show environmental persistence under anaerobic conditions (60, 175). Surfactants bound to the surface of soil or sediment particles (e.g., POEA) can be directly taken up by the filter-feeding aquatic invertebrates [e.g., water fleas (*Cladocera*)], soil organisms [e.g., earthworms (*Lumbricidae*) and springtails (*Collembola*)], and thus, can enter into the food chain (176). Moreover, OP and NP compounds and their ethoxylates have been detected even in human breast milk (177) indicating substantive human exposure.

## Conclusion

Residues of agrochemicals, e.g., VDs and PPP active ingredients, may reach food and feed products and through those can cause human, livestock, and environmental exposure. The rate of occurrence and the connectivity matrix of VDs and PPPs as contaminants in Europe are readily characterized by surveying notifications of contamination cases in the RASFF of the EU. Within such surveys, a comparative analysis of the numbers and trends in RASFF notifications for VDs and PPPs is rather informative. The identification cases of pesticide residues in the RASFF database are over 70% higher than that of VD residues: with 2,036 and 3,527 notifications for VDs and PPPs, respectively, between 2002 and 2016. Moreover, the two groups displayed opposing trends in time. Pesticide and VD residues rank 3^rd^ and 7^th^ in the overall notifications in RASFF, and the certainty in the RA status (obligatory to be assessed in RASFF since 2012) of the contamination cases is also more favorable for VDs than for pesticides. The initial high number of reported cases in 2002 for VD residues has successfully been pushed to a level below 100 cases annually by 2006. In contrast, the number of notification cases for pesticide residues shows a gradual increase from a low (approximately 50 cases annually) initial level until 2005, with a drop only in 2016, still representing over 250 cases annually. These opposing tendencies are explained by differing toxicology background in the two sectors, the assessment of VDs being deeply rooted in the evaluation of human pharmaceuticals. Yet, the fact that most commonly found VD residues to date are antibiotics remains to be a substantial concern.

Network analysis of connections between notifying and consigning countries reveal a Germany–Vietnam axis with main notifier countries being Germany, Belgium, the UK, and Italy (63, 59, 42, and 31 notifications announced, respectively) and main consigning countries of extensive non-compliances being Vietnam, India, China, and Brazil (88, 50, 34, and 23 notifications received, respectively). Thus, countries of South and Southeast Asia are considered a vulnerable point with regard to VD residues entering the EU market.

Toxicity problems may emerge not only due to the active ingredients but also due to additives used for formulation of veterinary pharmaceuticals and pesticides. During the production of VDs, feed additives, and PPPs, significant amounts of different surfactants are applied. Surfactants in VDs are mainly used as disinfectants, surface cleaning supplies, agents for animal bath, emulsifying and dispersing agents, emulsion stabilizers, and binders. In feed additives surfactants promote better digestibility and availability of nutrients. In pesticide formulations, the efficiency of the applied active ingredient is enhanced by the use of surfactants as adjuvants. Additives used for the production of preparations applied as VDs, animal feed supplements and PPPs according to the current regulation, are considered as inert or inactive ingredients (13).

According to current legislation, simpler ERA of additives is sufficient than the requirements for the active ingredients. Regulatory requirements, health RA, and ERA of active ingredients used in VDs are very strict, similar to the legal requisites regarding human medicines. In case of pesticide formulations, full toxicology tests are required for the active ingredient(s), but not for the formulated preparation. The determination of MRLs for VDs includes all components used in the veterinary preparations and vaccines with pharmacological or pharmacodynamic activity (12). In contrast, MRLs are set for pesticide active ingredients and their metabolites only and not for their adjuvants (17). In addition, the quantity of acceptable daily intake (ADI-value) of different formulations is typically determined on the basis of studies conducted with the active ingredient and not with the formulated preparations (152).

Recently, additive, synergistic, or antagonistic effects between the active ingredient(s) and additives, as well as individual toxicity of surfactants, have been demonstrated by several studies (106, 108, 152, 153). On the basis of the scientific evidence, the properties of these substances and their role in various biological interactions, these substances cannot be considered as unequivocally inactive ingredients by ecotoxicological and toxicological aspects in ERA of VDs, animal food supplements, and PPPs. Therefore, full toxicological assessment and evaluation of the adjuvants (e.g., surfactants) used in these formulated products is essential.

## Author Contributions

AS conceived the concept of the review. SK did the literature search, wrote the initial draft of the manuscript, and prepared the figures and tables. PB provided use and trade information, as well as physico-chemical data and descriptors of surfactants used in the formulation of VDs and PPPs. BD and AS oversaw the project, edited the manuscript, and took responsibility for the integrity of the data.

## Conflict of Interest Statement

The authors declare that the research was conducted in the absence of any commercial or financial relationships that could be construed as a potential conflict of interest.

## Abbreviations

ADBAC, dialkyl dimethyl ammonium chloride; AEO, alcohol ethoxylate; ALS, ammonium lauryl sulfate; AMOZ, 3-amino-5-morpholinomethyl-2-oxazolidone; ANEO, alkylamine ethoxylate; AOZ, 3-amino-2-oxazolidinone; APE, alkylphenol ethoxylate; APG, alkyl polyglycoside; CTAC, cetyl trimethyl ammonium chloride; DEA, diethanolamine; DSS, dioctyl sodium sulfosuccinate; EC, European Commission; ECHA, European Chemicals Agency; EFSA, European Food Safety Authority; EMA, European Medicines Agency; ERA, environmental risk assessment; EU, European Union; EURL, EU Reference Laboratory; GSEE, glycerol sorbitane ester ethoxylate; LAS, linear alkylbenzene sulfonate; MRL, maximum residue limit; MS, Member State; NP, nonylphenol; NRL, National Reference Laboratory; OP, octylphenol; PEC, predicted environmental concentration; PEG, polyethylene glycol; PNEC, predicted no-effect concentration; POEA, polyethoxylated tallow amine; PPP, plant protection product; QACs, quaternary ammonium compounds; RA, risk assessment; RASFF, Rapid Alert System for Food and Feed; REACH, Registration, Evaluation, Authorization and Restriction of Chemicals; SDS, sodium dodecyl sulfate; SEM, semicarbazide; SPC, specific product characteristics; TSE, transmissible spongiform encephalopathy; UK, United Kingdom; USA, United States of America; VD, veterinary drug.
